# Barriers and facilitators of medicines reconciliation at transitions of care in Ireland – a qualitative study

**DOI:** 10.1186/s12875-020-01188-9

**Published:** 2020-06-23

**Authors:** Patrick Redmond, Khalid Munir, Oludare Alabi, Tamasine Grimes, Barbara Clyne, Carmel Hughes, Tom Fahey

**Affiliations:** 1 HRB Centre for Primary Care Research, Royal College of Surgeons in Ireland, Dublin, Ireland; 2 School of Population Health and Environmental Sciences, King’s College London, London, UK; 3 School of Pharmacy and Pharmaceutical Sciences, Trinity College Dublin, Dublin, Ireland; 4 School of Pharmacy, Queen’s University Belfast, Belfast, Northern Ireland

**Keywords:** Medicines reconciliation, Patient safety, Qualitative research, Health plan implementation, Continuity of patient care/organization & administration

## Abstract

**Background:**

Medication error at transitions of care is common. The implementation of medicines reconciliation processes to improve this issue has been recommended by many regulatory and safety organisations. The aim of this study was to gain insight from healthcare professionals on the barriers and facilitators to the medicines reconciliation implementation process.

**Methods:**

Semi-structured interviews were conducted in Ireland with a wide range of healthcare professionals (HCPs) involved with medicines reconciliation at transitions of care. Thematic analysis was undertaken using an adaptation of a combined theoretical framework of Grol, Cabana and Sluisveld to classify the barriers and facilitators to implementation of medicines reconciliation.

**Results:**

Thirty-five participants were interviewed, including eleven community pharmacists (CPs), eight hospital pharmacists (HPs), nine hospital consultants (HCs), five general practitioners (GPs), and two non-consultant hospital doctors (NCHDs). Themes were categorized into barriers and facilitators. Barriers included resistance from existing professional cultures, staff interest and training, poor communication and minimal information and communications technology (ICT) support. Solutions (facilitators) suggested included supporting effective multidisciplinary teams, greater involvement of pharmacists in medicines reconciliation, ICT solutions (linked prescribing databases, decision support systems) and increased funding to provide additional (e.g. admission and discharge reconciliation) and more advanced services (e.g. community pharmacist delivered medicines use review).

**Conclusions:**

Medicines reconciliation is advocated as a solution to the known problem of medication error at transitions of care. This study identifies the key challenges and potential solutions that policy makers, managers and HCPs should consider when reviewing the practices and processes of medicines reconciliation in their own organisations.

## Background

Medication error during transitions of care can occur as a result of incomplete or inaccurate communication as responsibility shifts between healthcare providers or back to the patient and/or carer. Medication reconciliation is recommended by many patient safety and regulatory organisations as a process to reduce these errors [1–3].

Medicines reconciliation is defined as “the process of identifying the most accurate list of a patient’s current medicines—including the name, dosage, frequency, and route—and comparing them to the current list, recognizing discrepancies, and documenting any changes, thus resulting in a complete list of medications, accurately communicated” [4]. While regulatory organisations may require reconciliation, they are not specific in the mechanism required to undertake this. Indeed, a failure to agree practically useful outcomes, an ambiguity in intervention requirements and an unpreparedness for local circumstances suggest the need for an implementation science review of current practice [5, 6].

Many differing examples of implementation theories for healthcare interventions have previously been published [7]. The theories attempt to describe the complex and multiple influences on the success or failure in adopting a new process. These influences include the innovation itself, the receptiveness of actors within the system, organisational or system adoption of the innovation, networks of dissemination, and extra-organisational issues (e.g. socio-political). A number of previous studies have examined the experience of healthcare professionals (HCPs) (including physicians, nurses, pharmacists) and hospital administrators in managing medicines at transitions of care [8–14]. However, a systematic understanding of the factors that influence implementation of medicines reconciliation in Ireland is lacking.

The aim of this study was to explore the barriers and facilitators with healthcare professionals to the implementation of medicines reconciliation both between and within primary and secondary care in Ireland.

## Methods

A qualitative study was undertaken, with data being collected via face-to-face semi-structured interviews. A standardized reporting framework for qualitative studies was used (COREQ) (Supplementary Table 1) [15, 16].

### Research team and reflexivity

The first author (PR), a practising male GP, was a PhD student in Health Services Research interacting regularly with healthcare providers including some of the participants involved in this study. The research team (with backgrounds in pharmacy and health services research) identified the initial participants as per the sampling strategy (see below), with some participants known to the research team in advance and some suggested by participants during the study period. Beyond declaring an interest in the area of medication reconciliation, neither personal goals nor future research agendas were discussed with participants by the interview team.

### Theoretical framework

**Fig. 1:**
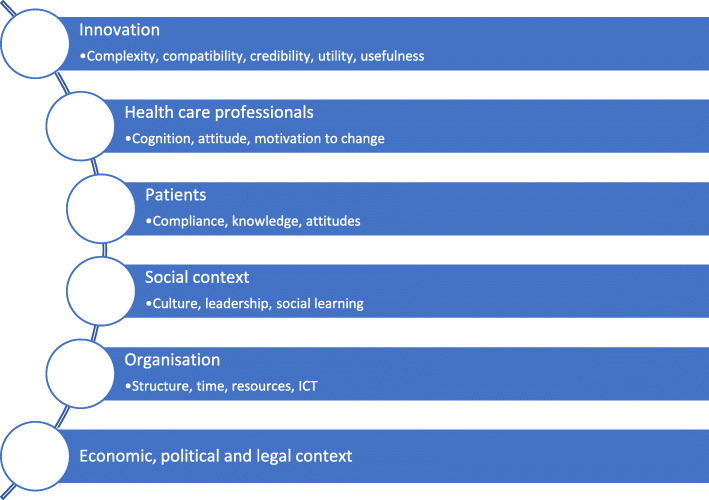
Barriers and drivers to the implementation of medicines reconciliation. Adapted from Sluisveld, 2012 [13]

The theoretical framework used, as shown in Fig. 1, was used to support categorisation of the identified barriers and facilitators to implementation of reconciliation. This model’s thematic structure is broadly similar to previously derived implementation models and allows easy comparison of our results with its application in previous studies’ settings [13] – both those specific to reconciliation and to healthcare interventions more generally.

### Context

Healthcare in Ireland has a mixed model of funding - most acute hospitals are publicly funded by the Health Service Executive (HSE) over four geographic regions (HSE Dublin-Mid Leinster, North East, South and West). Hospital consultants (HC) may practise in private and publicly funded institutions. Community pharmacists (CPs) and general practitioners (GPs) are private contractors who provide care to patients who are publicly funded as well as self-paying. Many different HCPs are involved in coordinating the care of patients both within and between primary and secondary care e.g. hospital-based physicians (both specialist and in training – non consultant hospital doctors - NCHDs), hospital pharmacists (HPs), CPs, and GPs. There is little interoperability within or between primary and secondary care systems. No institution provides comprehensive electronic prescribing. The majority of prescribing is done by doctors; prescriptions initiated in secondary care are often transcribed for longterm use by GPs in primary care. Medication errors at care transitions continue to be common [17–19].

### Sampling strategy

The target population was doctors and pharmacists directly involved with medicines reconciliation between primary and secondary care in Ireland. Purposive sampling was used to ensure maximum variation in terms of clinical commitment, geographic region, profession etc. The number of interviews depended on reaching thematic saturation across the group, which was determined by diminishing returns in concurrent data analysis [20, 21].

### Data collection methods & instruments

Data were collected between July and December 2015. Interviews took place in or near the respondents’ own offices/homes. Interviews, ranging 30–60 min in duration, were conducted using a semi-structured interview guide and audio recorded. The interview guide was devised based on existing literature, the theoretical framework (Fig. 1) and discussion with the research team (PR, TG, TF, CH) (Supplementary Table 2). Three members of the research team conducted the interviews (PR, OA, KM). All HCPs were interviewed alone. The interview questions consisted of closed, open-ended and probing questions (e.g., “Are adverse drug events a significant cause of morbidity/mortality for your patients?”; “What does medication reconciliation mean to you?”). Field notes may have been taken by the interviewer. While these were not the basis for analysis, interviewers were debriefed (PR) after each interview with field notes, where available, to identify additional potential areas of exploration, and focus for subsequent interviews.

### Data processing

Voice recordings were transcribed verbatim, with any identifying information removed. Only one author (PR) had access to the file linking transcripts with respondents’ identities. The transcription was checked against the original recording for accuracy. Transcriptions were returned to all participants for comments and/or correction (one participant clarified a response with additional detail) and imported into NVivo software for analysis [22].

### Data analysis

One researcher (PR) was primarily responsible for data entry, management and analysis with an additional researcher (BC) verifying a random sample of 10% for consistency of coding. A process of line-by-line deductive coding was undertaken. Research team members compared codes within and across interviews to elucidate themes. The essence of participants’ experiences was distilled through significant statements allied to the existing theoretical framework [13, 23]. Where data represented more than one theme, dual coding was undertaken. Where novel themes were uncovered that could not be placed within the existing framework, new codes were developed.

## Results

Sixty-one invitations were issued, 36 individuals consented, and 35 HCPs were interviewed (eleven CPs, eight HPs, nine HCs, five GPs, and two NCHDs) (Table 1). One consented HCP was uncontactable for the duration of the study.

**Table 1:** Characteristics of participants

Key Characteristics	*N* (%)
Participants	35
Gender	
Male	17 (49)
Female	18 (51)
Role	
Community Pharmacist	11 (31)
Hospital Pharmacist	8 (23)
Hospital Consultant:	9 (26)
• Medical specialty	5
• Emergency Medicine	1
• Acute Medical Assessment	1
• Anaesthetics	1
• Surgery	
o Ear Nose and Throat	1
General Practitioner	5 (14)
Non-Consultant Hospital Doctor	2 (6)
Health Service Executive (HSE) Region	
HSE Dublin Mid Leinster	27 (77)
HSE Dublin North East	2 (6)
HSE West	2 (6)
HSE South	4 (11)
Prescribing role	
Prescriber	16 (46)
Non-prescriber	19 (54)

Selected illustrative quotes from HCPs are listed below as examples of barriers and facilitators under each of the theoretical framework’s themes. It was not necessary to create additional main themes. The main points for each theme are summarised in Table 2, categorised under barriers and facilitators.

**Table 2:** Summary of themes describing barriers and driver to medicines reconciliation

	Barriers	Facilitators
Innovation	• Complex - many different healthcare providers • Poor existing communication pathways	• Tailoring processes to local needs • Standard operating procedures and staff adoption of same
Healthcare Professionals	• Staff training and supervision • Existing culture and hierarchies • Interest and awareness of reconciliation • Unclear lines of responsibility • Time pressures and prioritization	• Institutional effort to boost profile of reconciliation • Teaching prescribing • Culture change
Patients	• Lack of health literacy • Responsibility of prescribing information – patient vs HCP • HCP commitment to patient education	• Empowering patients • Risk stratifying/targeting those most at risk • Involving patient supports e.g. family members, ICT, multi-compartment compliance aids
Social context	• Multiple prescribers not communicating • Lack of effective multidisciplinary care (not supporting new roles, not sharing information)	• Clear, effective, systematic lines of communication • Teamwork culture • Local leaders, social learning and disseminating good practice
Organisation	• Lack of a coordinated ICT strategy • Fallible paper-based systems • System not robust enough to accommodate different patient presentations e.g. elective vs non-elective • Service availability not reflecting need • Lack of funding/remuneration to expand activities • Training, supervision, capacity of NCHDs all limited • HPs absent from hospital discharge • Clinical and prescribing information not intrinsically linked	• Funding to increase staff/service capability e.g. 8 am-8 pm, more FTEs • ICT solutions – linked prescribing databases, decision support systems • Greater involvement of pharmacists e.g. pharmacist prescribing, medicines use reviews
Political, legal and economic	• Ambiguity around official ‘MedRec’ policy • Disconnect between policy and practice • Discrepancy between private and publicly funded patients • Contractual/remuneration concerns • Data protection concerns	• Positive steps by health authority appointing health informatics lead • Putting in place systems to support good prescribing practice • Feedback on good/bad practice

*HCP* Healthcare Professional, *ICT* Information Communication Technology, *FTE* Full Time Equivalent, *NCHD* Non-consultant Hospital Doctor, *HP* Hospital Pharmacist

### Theme 1 – innovation

This theme addressed the complexity, feasibility and usefulness of the intervention. Implementing medicines reconciliation was described by most contributors as a complex process. The complexity of the reconciliation intervention and broader but related healthcare system issues (e.g. discharge communication, medicines management, prescribing competency, clinical supervision) was often overlapping and difficult to disentangle in the interviewees’ responses. This was highlighted in responses that listed the number of HCPs and sources (e.g. GP, carer, community pharmacy) that need to be consulted to conduct a comprehensive medicines reconciliation:

“*It is complex because of the number of people involved. So, you have invariably got the patient and their wider carers and family etc. You’ve got the community pharmacy, you’ve got the GP, you can have other services … so it’s not just one source …*” HP3

The established communication pathways between HCPs, and their failings, were underlined as barriers:

“*Often there are substantial delays in effective communication from one prescriber to the next and the information coming back from hospitals is not infrequently late, not infrequently illegible, not infrequently contains inaccuracies and all of that is a challenge*” GP3

The facilitators in implementing medicines reconciliation included tailoring the process to locally available resources:

*“I think it’s something that has to have a certain degree of fluidity to it and perhaps has to be a little bit localised in some centres … that’s appropriate to their resources, to their patient cohort and to the different interfaces they have with the community”* HP5

The strengths of certain staff in adapting to new procedures were recognised:

“*One of the key things to ours* [local reconciliation initiative] *was that it was nurse-led. We put a huge resource into nursing. Because nurses understand processes and they want to be told, ‘This is a standard operating procedure.’ You tell doctors that, they just think - They haven’t a clue what you’re talking about*” HC4

### Theme 2 - healthcare professionals

This theme encompasses issues of attitudes, motivation to change, knowledge and education. Indeed, staff training, across different disciplines and with the transient nature of some staff (e.g. NCHDs), was recognised as important but challenging to implement:

“*A lot of it, obviously, is education and trying to get education across to layers and layers of people in a healthcare setting … all who are changing over very frequently*” HC2

The culture specific to each profession was identified as a barrier to effective HCP teamwork:

“*We have a medical culture at the moment that imbues a certain level of autonomy to doctors … so they don’t want to be told by a pharmacist or a nurse that they’re doing the wrong thing … And nobody feeds back to them because they’re at the top of the profession*” HC4

HCPs’ responses were often not limited to medicines reconciliation and extended to discussions about patient safety and medicines management more generally. Low HCP interest in, and limited attention to, reconciliation and medicines management was a reported barrier:

“*The thing that frustrates me is my colleagues’ ambivalence … it needs to be from the top down. So, if the clinician leading out in an area doesn’t think it’s important, then their team is going to feel that it’s even less important*” HC1

To address these barriers, participants recommended empowering doctors in training to acknowledge a deficit in knowledge/training (or an opportunity for professional development) in prescribing:

“*A cultural change embodies a whole load of things. So, in other words, you’ll know you’ve succeeded when somebody’s entering their Day 1 as an intern and goes, ‘Excuse me, I just feel totally unprepared to address the prescribing issues in this hospital …*” HC4

Overlapping with social and organisational themes, respondents highlighted the institutional responsibility to increase the medicines reconciliation profile as a patient safety issue and to garner patient interest:

“*The Director of Quality, Safety and Improvement here is a consultant and the fact that medicines reconciliation is included in those guidelines means it is seen as more of a high-profile issue within the hospital which you would hope would help direct resources in that direction*” HP1

### Theme 3 - patients

This theme related to issues experienced by, or with direct input from, patients e.g. polypharmacy, multimorbidity, medication knowledge, attitude and adherence. Many responses in this theme were an interplay between the patient input and the organisational provisions for patients (e.g. patient own drug schemes, medicines information provision etc.).

Many participants underlined the perceived lack of interest by patients in their own medicines:

“*I think we have to try and get patients to be responsible for their own mediciness and I know with elderly patients it’s difficult but there’s a lot of people in that just don’t take responsibility*” CP11

Patients’ health literacy relating to mediciness was raised as a contributing factor in compiling an accurate medicines list:

“*There’s definitely, like, a patient empowerment issue, in that more better-off patients would come in with a very clear list … While other patients would come in and they would have blister packs and … they wouldn’t have much knowledge beyond that.”* HP7

One respondent felt the medicines administration process in their hospital led to difficulties for staff in empowering patients to take control of managing their own medicines following discharge:

“*I think there’s quite a bit of work to be done on understanding the control that the patient needs to be in, in order to function independently when they go home versus the level of control you need to have when the patient is in hospital*” HC3

Patient empowerment by education was identified as an opportunity:

*“We’re sending patients out of the hospital … and we’re not saying to them, ‘here’s a personal list of your medication and you have control over them … We* [need to] *empower the patient”* HP6

Targeting those patients more at risk of medication error, through morbidity or medicines burden, was deemed important, for example, cognitive decline and associated capacity issues relating to medicines management. Suggested strategies included involving family members in medicines management, and risk stratification on admission to hospital, or use of technology aids.

### Theme 4 - social context

This theme describes issues such as collaboration between colleagues, leadership, colleagues’ opinion and social learning. The many possible combinations of HCPs involved in a patient’s care, and their lack of communication, were raised as barriers to effective reconciliation:

“*Historically, I suppose the GP was very much in control of prescribing everything for a patient, whereas now they are being referred to different disciplines …*” CP6

The difficulty in building effective multidisciplinary teams, a proposed solution, was discussed. Different training, staff hierarchies or beliefs around healthcare delivery were seen as entrenched, especially between doctors and other HCPs:

*“I know the other consultants I work with; they don’t take kindly to anybody telling them what to do … It’s far better for the patient when we work together; it’s actually a far healthier dynamic”* HC6

The lack of interdisciplinary communication (in primary care) in clarifying medication regimens was raised by many contributors:

*“There’s no discussion of the medication between the pharmacist and the doctors. We’re not a primary care team here...we never sit down to discuss medication that certain patients are on so communication could be better”* CP8

Leading by example and social learning, for example involving all staff in the medicines reconciliation challenge, were listed as good practices: [24]

“*… we’ve gotten the consultants on board … the new service that we’re providing have bought into medicines reconciliation and recognise it as an important part of the admission, and look for it and ask for it in their patients”* HP2

### Theme 5 - organisation

This theme encompassed issues relating to existing care processes/structures, resources (time, staff, and capacity) and ICT infrastructure.

Frustration with ICT issues was frequently reported. Numerous examples were presented including incompatibility of handwritten and electronic systems, inaccurate electronic records, and lack of interoperability or coordination between and within settings.

There was a perceived lack of a coordinated national strategy to utilise electronic solutions to improve medicines management:

*“The way IT systems have been developed in hospitals has been a complete and utter disaster because everybody has bought a bit of equipment here and a bit of equipment there but none of the equipment talks to each other”* HC2

Handwritten and paper-based systems were singled out as sources of error:

*“...the system has got too complex to be operating at this level. We have complex medicines and we’re using a paper based system and paper communication and paper everything. It’s nonsense”* HP6

Many respondents reported that HPs were rarely involved in care delivery at hospital discharge:

*“We’re very aware at corporate level that there’s a need for MedRec at the point of discharge, not just at the point of admission. We just simply don’t have the resources to provide that at the minute”* HP1

Many respondents discussed the creation of new roles or the shifting of tasks from the traditional providers e.g. pharmacy technicians, prescribing pharmacists:

*“we need a third-tier … so that technicians can do more at the bedside and then the pharmacists can do more”* HP4

ICT was seen, by many, as a major component of an effective reconciliation programme. A linked accessible dispensing database was described by one contributor:

*“The thing that frustrates me most is information held in pockets. When I worked in* [internationally]*, we had an electronic patients’ record … I could link into their dispensing pharmacy and see what they had been dispensed and link it to compliance”* HC1

### Theme 6 - economic, political and legal issues

This theme covered political, legal and regulatory issues. The barriers to reconciliation listed here presented conflicting views from respondents. In particular, when asked to discuss guidelines in this area, respondents broadened their responses to reflecting on guidelines and legal responsibilities in general:

*"There isn’t any really … … formal guidelines that we, you know, have to adhere to. I suppose that may be part of the issue. So, I do think it is all a little bit* ad hoc*.* CP6

A consequence of Ireland’s mixed private-publicly funded healthcare is the difference in which prescribing information for self-paying patients is handled e.g. publicly funded patients have their hospital prescriptions transcribed by the GP prior to dispensing whereas private patients do not have this restriction. This discrepancy in prescription handling arises here:

*“There are plenty of private patients where you have no idea what medication they’re on because they don’t come to us very often as they don’t need to come to us to get the prescriptions done …*” GP4

Funding was a common topic relating to staff education, ICT systems, and local initiatives:

*“Resources are … a huge problem. There have been enormous cutbacks in every hospital … there’s only so far you can cut it back and still be safe. So, I think we’ve kind of got to that stage now”* HC2

Data protection concerns around sharing of electronic information were raised. Positive steps being taken by HSE were commended, such as appointing a health ICT lead.

## Discussion

### Summary

This study presents the views of key HCPs on the barriers and facilitators to medicines reconciliation in Ireland which were analysed using an implementation science theoretical framework. The most commonly reported barriers were organisation of care issues (e.g. ICT infrastructure), and the attitude and awareness of HCPs. The most frequently noted facilitators to effective medicines reconciliation were coded under the theme of social context (e.g. collaboration) and organisational issues such as the availability of ICT infrastructure.

The key findings centred on the themes of organisation of care, social context and healthcare professionals. Within these themes, both barriers and drivers were reported. Barriers reported included issues such as lack of electronic prescription databases, reliance on handwritten records, no interoperability between primary and secondary care ICT systems, staff attitudes and existing hierarchical structures. Potential solutions described the relationship between individuals and groups of individuals (i.e. teams, communication, and local leadership) as distinct from the hard infrastructure (i.e. ICT) or legal responsibilities.

Less commonly reported themes were discussions around patient health literacy, patients’ responsibility for their own medicines lists, HCPs’ responsibility to educate patients on their mediciness and discussion around the innovation (reconciliation) itself. The disconnect between primary and secondary care in terms of their funding sources (independent contractors versus publicly funded employees respectively) meant that while most respondents mentioned funding as an issue, the likely solutions in this area will be different between settings with CPs, for example, mentioning contractual negotiations to engage in medicines use reviews.

### Strengths and limitations

Our analysis of barriers and facilitators provided detailed information for professionals or organisations, regionally or nationally, to develop multifaceted implementation strategies for improving the implementation process of medicines reconciliation.

However, the sampling strategy in selecting interviewees, while purposive, was limited in the number of different kinds and geographic location of HCPs who participated, and this may limit the transferability of our findings. Nevertheless, the generated themes were common to the included professional groupings suggesting similar experiences (note only selected quotes are presented due to space constraints).

Interviewees may have been subject to social desirability bias. Additionally, due to the interpretative nature of qualitative research, the research team may have introduced confirmation bias [25]. The choice of data collection method may have been improved by triangulating the findings though alternative techniques (e.g. participant observation research) [23, 25]. Finally, the use of a pre-identified theoretical framework may have limited the potential breadth of responses. Nevertheless, the chosen model’s thematic structure is broadly similar to previously derived implementation models and allows easy comparison of our results with its application in previous studies’ settings [5–7, 13].

### Comparison with existing literature

The results of this study are similar to previously reported studies internationally [13, 26]. Organisational issues, including task substitution and the greater involvement of non-traditional HCPs (e.g. prescribing pharmacists) in the prescribing process is increasingly common and shown to be effective [27]. However, coupled with this, is the need to have functioning multidisciplinary teams – through openness to the opinion of others and willingness to compromise [12, 28].

The difficulty of staff engagement and training regarding medicines safety, where there is a fluid and constant changing staff profile (e.g. NCHDs), has been raised previously [14]. Designing ICT systems to support good practice was seen as key by many respondents e.g. decision support systems, connected prescribing databases and health information exchanges. This has good face validity and study evidence [29–31]. There is also a recognition that the implementation of ICT is slow, dependent on local circumstances/complex communication arrangements, unrealistic expectations that are often hindered by conflicting strategic initiatives, and lack of immediately discernible benefits [32].

Effective reconciliation may also be hampered by increasing specialisation, where in some cases, physicians only consider their own specialist medicines; this can make it difficult to clarify with a prescriber the intent of prescription changes [33]. Coupled with the difficulty in integrating non-medical professionals into multidisciplinary teams, this can impede questioning about prescribing decisions and reduce the effectiveness of the team.

### Implications for research and practice

Future reconciliation interventions could be implemented through process mapping and feedback studies [e.g. Plan-Do-Study-Act (PDSA)] to specifically target the areas identified in this study. Policy makers should note the need for integrated solutions - many contributors focused on intra-organsational concerns but the patient’s journey is trans-organisational. In particular the integration of sociotechnical themes (interaction between organisational structures/processes and people working within them) seems likely to be most benefical [26, 34].

Future research should consider the overlap in examining topics such as medicines reconciliation, management, staff training, patient and organisational responsibility. The opinions of patients, nurses, carers and administrators also needs to be researched [35].

## Conclusion

Medicines reconciliation is advocated as a solution to the known problem of medicines discrepancies at transitions of care. This study identifies the key challenges and potential solutions that health policy makers, managers and HCPs in Ireland should consider when reviewing the practices and processes of medicines reconciliation in their own organisations. Key areas to focus on include staff support and training, effective multidisciplinary teams, greater involvement of pharmacists in medicines reconciliation, ICT solutions (linked prescribing/dispensing databases, decision support systems) and increased funding to provide additional (e.g. admission and discharge reconciliation) and more advanced services (e.g. dedicated CP delivered medicines reconciliation and medicines use review).

## Supplementary information

**Additional file 1.**

## Data Availability

The datasets generated during and/or analysed during the current study are available from the corresponding author on reasonable request.

## Abbreviations

CP: Community pharmacist
GP: General Practitioner
HC: Hospital Consultant
HP: Hospital Pharmacist
HCP: Healthcare professional
HSE: Health Service Executive
ICT: Information communication technology
NCHD: Nonconsultant Hospital Doctor
PDSA: Plan Do Study Act

## References

[1] World Health Organization (WHO) (2006). WHO High 5’s Project.

[2] National Centre for Health and Care Excellence (2015). Medicines optimisation: the safe and effective use of medicines to enable the best possible outcomes.

[3] Institute for Healthcare Improvement. How-to Guide: Prevent Adverse Drug Events by Implementing Medication Reconciliation. Cambridge: Institute for Healthcare Improvement; 2011. Available at https://www.ihi.org.

[4] Aspden P, Wolcott J, Bootman J, Cronenwett L (2006). Preventing medication errors.

[5] Damschroder LJ, Aron DC, Keith RE, Kirsh SR, Alexander JA, Lowery JC (2009). Fostering implementation of health services research findings into practice: a consolidated framework for advancing implementation science. Implement Sci.

[6] Grol R, Wensing M (2004). What drives change? Barriers to and incentives for achieving evidence-based practice. Med J Aust.

[7] Greenhalgh T, Robert G, Macfarlane F, Bate P, Kyriakidou O (2004). Diffusion of innovations in service organizations: systematic review and recommendations. Milbank Q.

[8] Boockvar KS, Santos SL, Kushniruk A, Johnson C, Nebeker JR (2011). Medication reconciliation: barriers and facilitators from the perspectives of resident physicians and pharmacists. J Hosp Med.

[9] Manias E, Gerdtz M, Williams A, McGuiness J, Dooley M. Communicating about the management of medications as patients move across transition points of care: an observation and interview study. J Eval Clin Pract. 2016;22 n/a-n/a. 10.1111/jep.12507 26762967

[10] Vogelsmeier A, Pepper GA, Oderda L, Weir C (2013). Medication reconciliation: a qualitative analysis of clinicians’ perceptions. Res Social Adm Pharm.

[11] Urban R, Paloumpi E, Rana N, Morgan J (2013). Communicating medication changes to community pharmacy post-discharge: the good, the bad, and the improvements. Int J Clin Pharm.

[12] Sanchez SH, Sethi SS, Santos SL, Boockvar K (2014). Implementing medication reconciliation from the planner’s perspective: a qualitative study. BMC Health Serv Res.

[13] van Sluisveld N, Zegers M, Natsch S, Wollersheim H (2012). Medication reconciliation at hospital admission and discharge: insufficient knowledge, unclear task reallocation and lack of collaboration as major barriers to medication safety. BMC Health Serv Res.

[14] Coffey M, Cornish P, Koonthanam T, Etchells E, Matlow A (2009). Implementation of admission medication reconciliation at two academic health sciences centres: challenges and success factors. Healthc Q.

[15] Tong A, Sainsbury P, Craig J (2007). Consolidated criteria for reporting qualitative research (COREQ): a 32-item checklist for interviews and focus groups. Int J Qual Health Care.

[16] O’Brien BC, Harris IB, Beckman TJ, Reed DA, Cook DA (2014). Standards for reporting qualitative research: a synthesis of recommendations. Acad Med.

[17] Grimes T, Duggan C, Delaney T (2010). Pharmacy services at admission and discharge in adult, acute, public hospitals in Ireland. Int J Pharm Pract.

[18] Grimes TC, Duggan C, Delaney TP, Graham IM, Conlon KC, Deasy E (2011). Medication details documented on hospital discharge: cross-sectional observational study of factors associated with medication non-reconciliation. Br J Clin Pharmacol.

[19] Riordan CO, Delaney T, Grimes T (2016). Exploring discharge prescribing errors and their propagation post-discharge: an observational study. Int J Clin Pharm.

[20] Ritchie J, Lewis J, Nicholls CM, Ormston R (2014). Qualitative research practice. A guide for social science students and researchers.

[21] Mason M. Sample size and saturation in PhD studies using qualitative interviews. Forum Qual Sozialforsch. 2010;11:3. 10.17169/fqs-11.3.1428.

[22] QSR (2012). NVivo qualitative data analysis software.

[23] Creswell JW (2012). Qualitative inquiry and research design: choosing among five approaches.

[24] Bandura A (1977). Social learning theory.

[25] Mays N, Pope C (1995). Qualitative research: observational methods in health care settings. BMJ..

[26] Linden-Lahti C, Holmström A-R, Pennanen P, Airaksinen M (2019). Facilitators and barriers in implementing medication safety practices across hospitals within 11 European Union countries. Pharm Pract (Granada).

[27] van den Bemt PM, van den Broek S, van Nunen AK, Harbers JB, Lenderink AW (2009). Medication reconciliation performed by pharmacy technicians at the time of preoperative screening. Ann Pharmacother.

[28] Machen S, Jani Y, Turner S, Marshall M, Fulop NJ. The role of organizational and professional cultures in medication safety: a scoping review of the literature. Int J Qual Heal Care. 2019;31:G146–57. 10.1093/intqhc/mzz111 PMC7097989 31822887

[29] Bates DW, Leape LL, Cullen DJ, Laird N, Petersen LA, Teich JM (1998). Effect of computerized physician order entry and a team intervention on prevention of serious medication errors. JAMA..

[30] Cresswell K, Bates DW, Sheikh A (2016). Six ways for governments to get value from health IT. Lancet..

[31] Bates DW, Kuperman GJ, Wang S, Gandhi T, Kittler A, Volk L (2003). Ten commandments for effective clinical decision support: making the practice of evidence-based medicine a reality. J Am Med Informatics Assoc.

[32] Sheikh A, Cornford T, Barber N, Avery A, Takian A, Lichtner V (2011). Implementation and adoption of nationwide electronic health records in secondary care in England: final qualitative results from prospective national evaluation in “early adopter” hospitals. BMJ..

[33] Pevnick JM, Shane R, Schnipper JL. The problem with medication reconciliation. BMJ Qual Saf. 2016;25:726–30. 10.1136/bmjqs-2015-004734 PMC4956589 26795914

[34] Fox WM (1995). Sociotechnical system principles and guidelines: past and present. J Appl Behav Sci.

[35] Garfield S, Furniss D, Husson F, Etkind M, Williams M, Norton J, et al. How can patient-held lists of medication enhance patient safety? A mixed-methods study with a focus on user experience. BMJ Qual Saf. 2020;:bmjqs-2019-010194. 10.1136/bmjqs-2019-010194. 10.1136/bmjqs-2019-010194 PMC7467504 31949006

