# Supplementary material for: Barriers and facilitators of medicines reconciliation at transitions of care in Ireland – a qualitative study
Source: BMC Fam Pract. 2020 Jun 23;21:116. doi: 10.1186/s12875-020-01188-9 (PMC7313163; doi:10.1186/s12875-020-01188-9)
Supplement: Supplementary file 1 — Additional file 1. [file 12875_2020_1188_MOESM1_ESM.pdf]

Supplementary Table 1: COREQ Checklist

Consolidated criteria for reporting qualitative studies (COREQ): 32-item checklist

Developed from:

Tong A, Sainsbury P, Craig J. Consolidated criteria for reporting qualitative research (COREQ): a 32-item checklist for interviews and focus groups. *International Journal for Quality in Health Care*. 2007. Volume 19, Number 6: pp. 349 – 357

**No. Item**	**Guide questions/description**	**Reported on Page #**
**Domain 1: Research team and reflexivity**		
Personal Characteristics		
1. Inter viewer/facilitator	Which author/s conducted the inter view or focus group?	Page 4
2. Credentials	What were the researcher’s credentials? E.g. PhD, MD	Page 1
3. Occupation	What was their occupation at the time of the study?	Page 1 and 4
4. Gender	Was the researcher male or female?	Page 4
5. Experience and training	What experience or training did the researcher have?	Page 4
Relationship with participants		
6. Relationship established	Was a relationship established prior to study commencement?	Page 4 .

7. Participant knowledge of the interviewer	What did the participants know about the researcher? e.g. personal goals, reasons for doing the research	Page 4
8. Interviewer characteristics	What characteristics were reported about the inter viewer/facilitator? e.g. Bias, assumptions, reasons and interests in the research topic	Page 4

**Domain 2: study design**		
Theoretical framework		
9. Methodological orientation and Theory	What methodological orientation was stated to underpin the study? e.g. grounded theory, discourse analysis, ethnography, phenomenology, content analysis	Page 4
Participant selection		
10. Sampling	How were participants selected? e.g. purposive, convenience, consecutive, snowball	Page 5
11. Method of approach	How were participants approached? e.g. face-to-face, telephone, mail, email	Page 5
12. Sample size	How many participants were in the study?	Page 5
13. Non-participation	How many people refused to participate or dropped out? Reasons?	Page 5
Setting		
14. Setting of data collection	Where was the data collected? e.g. home, clinic, workplace	Page 5
15. Presence of non-participants	Was anyone else present besides the participants and researchers?	Page 5
16. Description of sample	What are the important characteristics of the sample? e.g. demographic data, date	Page 5 & Table 1
Data collection		

17. Interview guide	Were questions, prompts, guides provided by the authors? Was it pilot tested?	Appendix A and page 5
18. Repeat interviews	Were repeat inter views carried out? If yes, how many?	Page 5
19. Audio/visual recording	Did the research use audio or visual recording to collect the data?	Page 5
20. Field notes	Were field notes made during and/or after the inter view or focus group?	Page 5
21. Duration	What was the duration of the inter views or focus group?	Page 5
22. Data saturation	Was data saturation discussed?	Page 5
23. Transcripts returned	Were transcripts returned to participants for comment and/or correction?	Page 5
**Domain 3: analysis and findings**		
Data analysis		
24. Number of data coders	How many data coders coded the data?	Page 5
25. Description of the coding tree	Did authors provide a description of the coding tree?	Page 6
26. Derivation of themes	Were themes identified in advance or derived from the data?	Page 6
27. Software	What software, if applicable, was used to manage the data?	Page 5
28. Participant checking	Did participants provide feedback on the findings?	Page 5

Reporting		
29. Quotations presented	Were participant quotations presented to illustrate the themes/findings? Was each quotation identified? e.g. participant number	Page 6 to 10
30. Data and findings consistent	Was there consistency between the data presented and the findings?	Yes, there was.<br>Page 6 to 12
31. Clarity of major themes	Were major themes clearly presented in the findings?	Yes. they were.<br>From page 6 to 12
32. Clarity of minor themes	Is there a description of diverse cases or discussion of minor themes?	Discussion of major and minor themes<br>From page 6 to 12

Supplementary Table 2: Topic Guide

The overall goal of this research study is to improve medication reconciliation (MEDREC). If you have not had time to read the study information sheet, please do so now. Following this please review the consent sheet and, if you are happy to proceed, please sign it.<br><br>MEDREC is often defined as the processes that are used to ensure that patients and those who care for them have a good idea of what medications the patient is actually taking. This is particularly relevant when patients move between different healthcare providers and different intensities of health care provision, for example between hospital and their GP or between the ward and the ICU.<br>The purpose of this project is to provide both a wide and a detailed exploration of the barriers and drivers to the implementation of MEDREC between primary and secondary care in Ireland.<br><br>We are looking for your honest feelings and opinions; there are no right or wrong answers in this interview and all your responses will be confidential. In any reports of these interviews, we will never mention your name, practice, or any other personal information. We would also ask that you do not mention any third parties by name, like patient names etc.<br>Do you have any questions before we begin?"<br>Organisation & Healthcare professionals:<br>So, to begin, would you like to tell me a little bit about the environment you work in and your experience in dealing with the MEDREC process? [E.g. hospital admission, discharge; re-admission to community or information transfer b/w GP/CP etc.]<br>"What does medication reconciliation mean to you?"<br>Do you care for patients who experience a transition of care (TOC)?<br>Listen for description of complexity/good management.<br>Is this a complex process? What kinds of issues arise? What type of errors have you seen?<br>So, in your practice, are there any facilities like computers or regular audits that enable a more efficient reconciliation process or is anyone in particular assigned to making this process more efficient? [Does somebody collect all prescription scripts and sort them or is this down to individual GPs/pharmacists etc.?]<br>What to you are the barriers to effective MEDREC?<br>And conversely what supports it?<br>Do you actively involve yourself with reconciling patients' medication?<br>Are adverse drug events a significant cause of morbidity/mortality for your patients?"<br>Have you seen errors in patients' medication at points of transitions of care? How dangerous, in your opinion, were these errors? Is it a serious issue?<br>What do you see as the role of the... (Discharging doctor/pharmacist/community pharmacist/patient/GP)?<br>Do you think your role in the reconciliation process helps in preventing hospital admissions? Or providing for a safe admission? Or supports safe medication use after discharge?<br>Are adverse drug events a significant cause of morbidity/mortality for your patients, especially when thinking of MEDREC? [If gives an example - "How dangerous or severe in your opinion?"]<br>Do you note that certain medications are subject to errors more than others?<br>Social context:<br>What is the importance placed on reconciliation where you work?<br>Do you liaise with other GPs or pharmacists?<br>What support do you receive in managing patients' medications? Is this something that is discussed amongst your fellow GPs/pharmacists/nurses

Do you think, in your role and practice location, you are best placed to help patients in organising their medications? Do you feel that patients feel this is a necessary component of their care?
What, if any, are the unique challenges you face because of the socioeconomic factors, of where you work? E.g. literacy, income, chronic disease, attendance/follow-up issues?
Economic context:
Do you feel MEDREC is a big part of your job? If so, do you feel like this affects already limited time constraints and billing practices? [For example, Doctor-Patient interaction; private/public; practice requires seeing a certain no. of patients.]
Do you think remuneration would help to implement MEDREC?
Do you think you have sufficient resources to effectively carry out MEDREC? [Staff; Computers; Time; Funding]
What are the constraints economically? Are there issues that supersede reconciliation in your practice? [E.g. are there commercial or human resource issues that are more pressing?]
Political and legal context:
I would now like to discuss the role of health regulatory or government agencies in the area of MEDREC, in particular any guidelines you may be aware of or responsibilities you may have as a healthcare provider.
Are you aware of any guidelines relating to MEDREC? Are you aware of any responsibilities you may have as a healthcare provider?
The HSE quality and patient safety guidelines and HIQA have noted the importance of implementing MEDREC. Do you feel that this information is freely available?
Do you think there is much support from the HSE/Department of Health/Local Health authority for this issue?
Do you feel under any legal obligation to perform activities to make medication use safer at care transitions? Can you tell me about that?
Innovation:
How would you improve the process of MEDREC in your area? Do you feel it requires communication with too many parties? Do you think can be an efficient system for HCPs?
"Thank you. Is there anything else you would like to say about anything we discussed today? Thank you for your time and participation. Your input is extremely valuable; you will have an opportunity to review your transcript prior to final submission.
